# Effect of wild-type vaccine doses on BA.5 hybrid immunity, disease severity, and XBB reinfection risk

**DOI:** 10.1128/jvi.01285-24

**Published:** 2024-11-05

**Authors:** Daxiang Chen, Weihong Zhang, Bin Xiao, Banglao Xu, Xiaoyun Yang, Shidong Deng, Guichang Li, Gang Yang, Jinpeng Cao, Xinyue Mei, Qi Luo, Peiyu Huang, Xi Sun, Jie su, Nanshan Zhong, Zhuxiang Zhao, Zhongfang Wang

**Affiliations:** 1 State Key Laboratory of Respiratory Disease, National Clinical Research Center for Respiratory Disease, Guangzhou Institute of Respiratory Health, The First Affiliated Hospital of Guangzhou Medical University, Guangzhou Medical University, Guangzhou, Guangdong, China; 2 Hetao Institute of Guangzhou National Laboratory, Shenzhen, Guangdong, China; 3 Department of Laboratory Medicine, Guangzhou First People’s Hospital, Guangzhou Medical University, Guangzhou, China; 4 Department of Laboratory Medicine, The Affiliated Qingyuan Hospital (Qingyuan People’s Hospital), Guangzhou Medical University, Qingyuan, Guangdong, China; 5 Department of Infectious Disease, Respiratory and Critical Care Medicine, Guangzhou First People’s Hospital, Guangzhou Medical University, Guangzhou, China; Loyola University Chicago - Health Sciences Campus, Maywood, Illinois, USA

**Keywords:** SARS-CoV-2, vaccine, neutralizing antibodies, COVID-19 severity, vaccine dose

## Abstract

Vaccination against the wild-type (WT) severe acute respiratory syndrome coronavirus 2 (SARS-CoV-2) virus did not produce detectable levels of neutralizing antibodies (NAbs) against the BA.5 strain before it emerged. However, coronavirus disease-2019 (COVID-19) severity varied highly between unvaccinated, partially vaccinated, and fully vaccinated individuals, for unknown reasons. We assessed the severity of BA.5 infection and the risk of XBB strain reinfection and measured serum levels of NAbs against WT, BA.5, and XBB.1.9.1 SARS-CoV-2 strains at varying time points in 1,373 individuals who received zero, one, two, or three WT vaccine doses. We found that two to three WT doses significantly increased WT and BA.5 NAb levels and reduced the incidence of COVID-19-associated pneumonia upon BA.5 strain infection compared to zero to one dose. Regarding XBB reinfection, those who received two to three doses and were infected with the BA.5 variant exhibited a significantly lower reinfection risk compared to those who received zero to one dose. RNA analysis revealed that the differentially expressed genes between the two to three dose and unvaccinated groups were enriched in B cell activation, cytokine-cytokine receptor interaction, complement, and monocyte activation functions—indicating that vaccination increased the antibody response and reduced inflammation. Our results suggest that multiple antigen exposures to either matched or unmatched SARS-COV-2 variants, through vaccination or infection, may be necessary to achieve significant immune imprinting.

**IMPORTANCE:**

The administration of coronavirus disease-2019 (COVID-19) vaccines that do not perfectly match the viral strains that individuals become infected with has been found to impact the resultant illness severity—although the precise mechanism underlying this phenomenon remains unclear. We assessed viral clearance, as well as serum levels of inflammatory cytokines and neutralizing antibodies (NAbs) against wild-type, BA.5, and XBB.1.9.1 variants of the severe acute respiratory syndrome coronavirus 2 among individuals who received varying doses of such strain-mismatched vaccines. Notably, vaccination with ≥2 doses of strain-mismatched COVID-19 vaccines appeared to stimulate the production of specific NAbs during infection with new variants, as well as attenuate the inflammatory response and enhance viral clearance. Such vaccination regimens can also reduce the risk of reinfection. These findings may be important for guiding the development of future COVID-19 vaccination strategies that target both matched and mismatched viral variants.

## INTRODUCTION

As of 26 November 2023, 3.52 billion doses of coronavirus disease-2019 (COVID-19) vaccines have been administered in China, resulting in ~90% of the total population being covered by at least one dose (source: https://covid19.who.int). Moreover, ~87% of the Chinese population have completed the primary series of COVID-19 vaccinations and ~57% have received at least one booster dose. The primary vaccine series predominantly used inactivated viral particles of the wild-type (WT) strain of the severe acute respiratory syndrome coronavirus 2 (SARS-CoV-2). This was the case for the popular CoronaVac and BIBIBP-CorV vaccines, which together account for >95% of the administered vaccine doses in China (1). Either homologous or heterologous booster vaccines (such as ZF2001 and Convidecia) were later administered to many vaccinated adults ≥6 months after the primary vaccination series.

Vaccination with varying doses of the WT COVID-19 vaccine provides distinct levels of protection against different SARS-CoV-2 strains. The CoronaVac vaccine demonstrated an efficacy of 83.5% in preventing symptomatic COVID-19 infection against the dominant variants prior to the emergence of the Delta strain, 14 days or more following the administration of a second dose (2). Meanwhile, the subunit SARS-CoV-2 vaccine (ZF2001) demonstrated a 75.7% efficacy against infection with the Delta variant, and 87.6% against severe-to-critical COVID-19, at least 7 days following the administration of a third dose (3). Infections caused by the Omicron BA.2 variant, which surpassed the Delta variant as the dominant SARS-COV-2 strain during the recent COVID-19 pandemic, showed a significant inverse correlation between vaccination doses and clinical severity—with the risk of severity decreasing significantly at ≥21 days following the administration of a third vaccine dose (4). However, the administration of one, two, and three doses of the WT vaccine demonstrated efficacy rates of 0%, 17%, and 22%, respectively, for preventing infection with the BA.2 SARS-CoV-2 variant (1). These findings may be attributable to unique mutations present in the Omicron variant that affect its receptor binding, antibody evasion, and infectivity (5, 6). These qualities caused a significant reduction in the effectiveness of vaccine-induced immunity against SARS-CoV-2 infection when vaccinated individuals were challenged with the BA.1 or BA.2 strains (1, 3, 7). The persistent mutation of SARS-CoV-2 and its increasing divergence from the WT strain have raised concerns regarding the efficacy of WT vaccines against infections by new variants (8–10).

The novel BA.5 SARS-CoV-2 sub-lineage, characterized by additional mutations in the spike protein receptor-binding domain compared to the BA.2 one, has dominated the subsequent COVID-19 pandemic waves. Low levels of BA.5 neutralizing antibodies (NAbs) were found to be induced by three doses of vaccine (11). Furthermore, the BA.5 variant was found to evade NAbs in vaccinated individuals (including those who received booster doses) and patients infected with either the BA.1 or BA.2 strains (12–16). Moreover, little is known regarding the effects of vaccination against SARS-CoV-2 strains before BA.2, which was still present when BA.5 emerged. Wang et al. (17) observed that lower levels of NAbs against WT and BA.5 were induced in unvaccinated individuals who were infected with BA.5 or BF7 compared to those who were vaccinated with two or three doses, as determined by pseudovirus neutralization assays performed at 28 days following infection in 134 participants; moreover, more doses did not improve the NAb titer against the XBB strain. However, Guan et al. (18) conducted pseudovirus neutralization assays for 303 recovered patients who had been previously infected with the BA5 or BF7 variant and found no significant differences in NAbs against the SARS-CoV-2 WT strain or Omicron variant between those who received one to two doses vs who received three to four. Considering the potential influence of the pseudovirus neutralization assays and sample size on the outcomes, the impacts of different vaccine doses on the generation of NAbs following BA.5 infection merit elucidating. Notably, the effects of vaccination doses on the severity of BA.5 infection, speed of NAb recall (as well as its breadth and duration), and the potential reinfection risk of these vaccinated individuals against emerging strains (such as XBB) remain unclear.

In this study, we evaluated 1,606 samples collected from patients who were infected with the BA.5 variant and had been previously vaccinated with zero, one, two, or three doses of the WT vaccine, in order to determine the effect of vaccination doses on antibody responses and disease symptom characteristics against SARS-CoV-2 BA.5 variant. We also calculated the reinfection rates of 843 individuals at one hospital who received different doses of the WT COVID-19 vaccine prior to being infected with BA.5 over 1 week during the XBB wave of the pandemic. Through this, we aimed to elucidate the underlying mechanism of vaccine doses on immunity against the BA.5 variant, disease severity protection, and subsequent XBB reinfection risk, in an effort to guide future vaccine formulations and strategies.

## RESULTS

### WT COVID-19 vaccination mitigates the severity of COVID-19 attributable to the BA.5 strain in a dose-dependent manner

**Fig 1:**
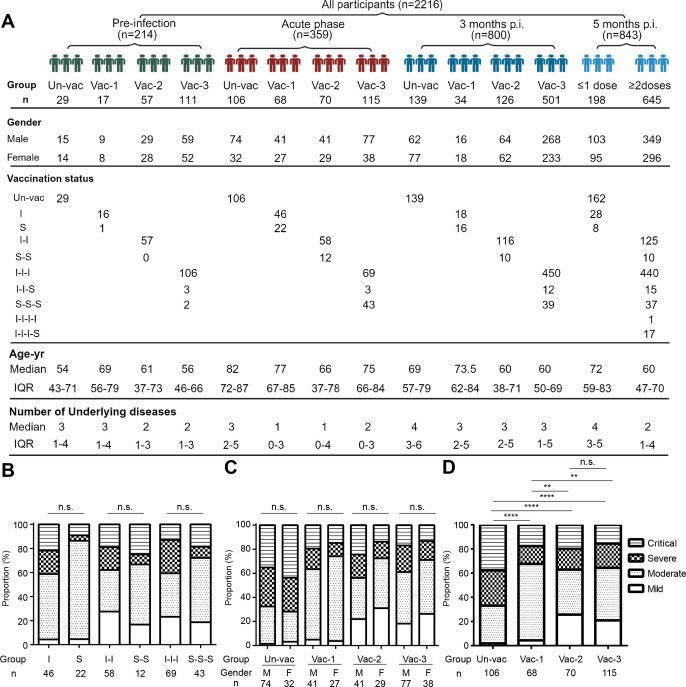
Characteristics of the four patient cohorts and the levels of COVID-19 severity during the acute disease phase. (**A)** Schematic representation of the study participants—including at the pre-infection, acute phase, and 3- and 5-months post-infection. The study participants were categorized based on vaccination doses. Characteristics such as sex, age, vaccination status, and underlying health conditions were documented. (**B)** The proportion of disease severity observed in the inactivated vaccine group compared to that in the S protein vaccine group. (**C)** The proportion of disease severity observed among the males and females. (**D)** The proportion of disease severity in the participants who had been vaccinated with zero, one, two, or three doses. The counts were analyzed using the Chi-squared test with a statistical significance level set at *P* < 0.05 (*P < 0.05; **P < 0.01; ***P < 0.001; ****P < 0.0001; and n.s., not significant.). Un-vac, unvaccinated individuals; Vac-1, individuals who had received one dose of COVID-19 vaccine; Vac-2, individuals who had received two doses of COVID-19 vaccine; Vac-3, individuals who had received three doses of COVID-19 vaccine. I, inactivated SARS-CoV-2 vaccine; S, RBD recombinant subunit vaccine; IQR, interquartile range.

The baseline characteristics of the participants are presented in Fig. 1A. We conducted analyses to investigate the impact of inactivated virus and S protein-based vaccines on the severity of COVID-19 caused by the SARS-CoV-2 BA.5 variant during the acute phase. Our results indicated that the two vaccine types, when administered at equivalent doses, did not show significant differences in terms of disease severity (Fig. 1B). Furthermore, no significant differences were observed in terms of disease severity between females and males who received identical doses (Fig. 1C).

To investigate whether different vaccination doses influenced the severity of COVID-19 induced by the BA.5 variant, we conducted a study assessing disease severity among 359 patients who had received zero, one, two, or three vaccine doses prior to becoming infected with the BA.5 variant (Fig. 1D). The incidence of severe or critical illness was lower (*P* < 0.0001) among vaccinated individuals (Vac-1, Vac-2, and Vac-3) than among unvaccinated ones (Un-vac), and the proportion of mild illness was higher in those who received ≥2 doses than in those who received only one (*P* = 0.001). However, no significant difference (*P* = 0.673) was observed in terms of disease severity between individuals who received two vs three doses. These data suggested that vaccination against WT SARS-CoV-2 mitigated the severity of COVID-19 attributable to BA.5 and that ≥2 vaccine doses significantly reduced the risk of developing pneumonia.

### Two or three WT vaccine doses induced more rapid increases in anti-WT and anti-BA.5 NAb levels following BA.5 infection

**Fig 2:**
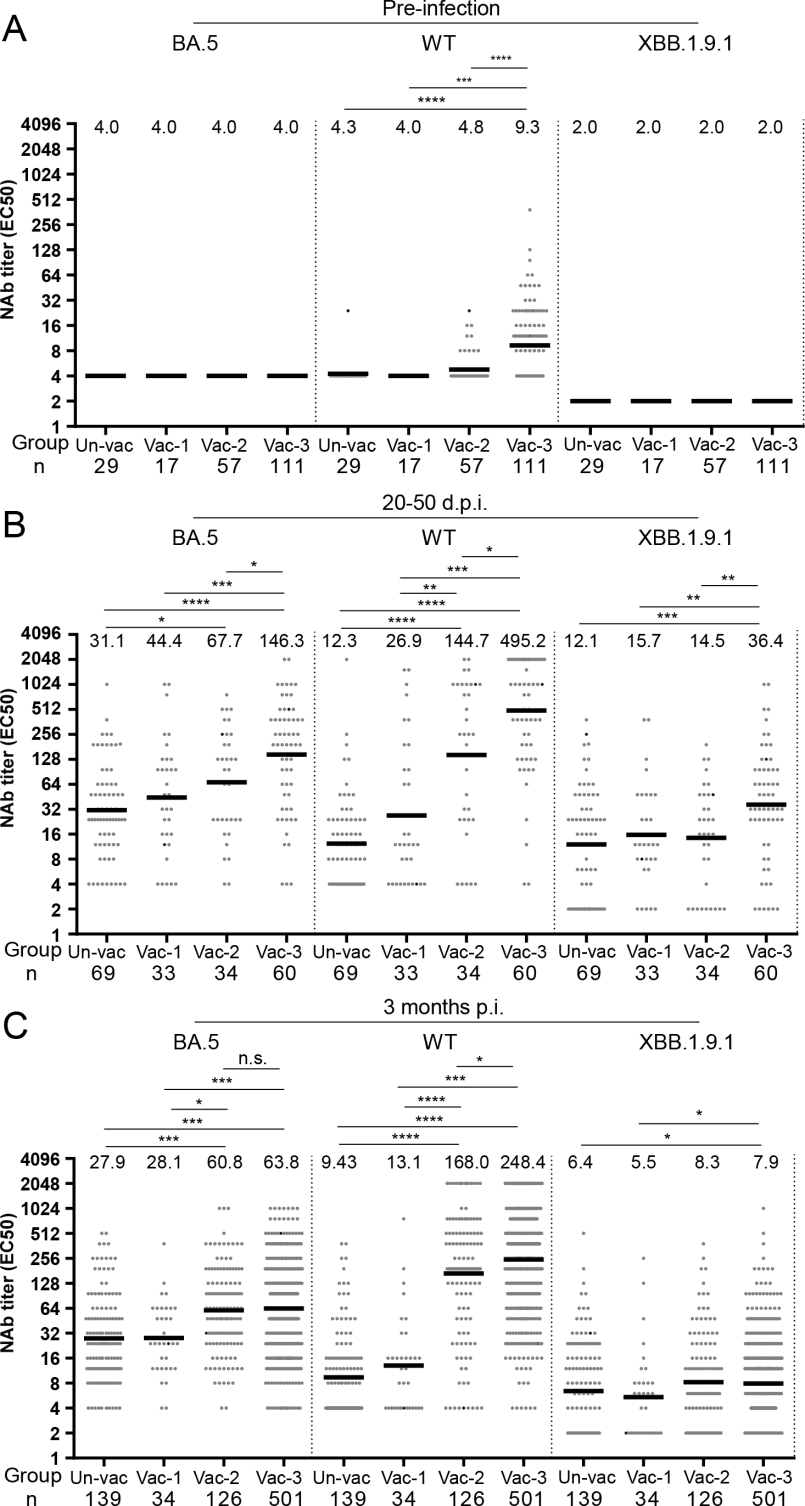
Neutralizing antibody titers against BA.5, WT, or XBB.1.9.1 in individuals vaccinated with zero, one, two, and three doses. (**A)** NAb titers against BA.5, wild-type, or XBB.1.9.1 were measured in individuals who had received zero, one, two, or three doses of the COVID-19 vaccine and had no prior SARS-CoV-2 infections. (**B)** NAb titers against BA.5, WT, or XBB.1.9.1 were assessed in individuals who had received zero, one, two, or three doses of the COVID-19 vaccine 20–50 days after being infected with the BA.5 variant. (**C)** NAb titers against BA.5, WT, or XBB.1.9.1 were assessed in individuals who had received zero, one, two, or three doses of the COVID-19 vaccine 3 months after being infected with the BA.5 variant. The NAb titers in each group are presented as geometric mean titers at the top of each panel. Significance was determined using Mann-Whitney *U* tests (*P < 0.05; **P < 0.01; ***P < 0.001; ****P < 0.0001; and n.s., not significant.).

Although WT vaccination could not induce anti-BA.5 NAbs even at 14 and 30 days after vaccination (11), it remained unclear whether it could affect infection-induced NAbs against the BA.5 and subsequent XBB strains. We, therefore, measured the patients’ NAb titers against the WT, BA.5, and XBB1.9.1 strains before and after BA.5 infection (Fig. 2). Two months before the BA.5 outbreak, those who received three doses of WT vaccine had significantly higher levels of anti-WT NAbs than those who received zero to two doses (Fig. 2A). However, anti-BA.5 and anti-XBB1.9.1 NAb levels were both below the detectable limit in all of the groups (Fig. 2A).

At 20–50 days post-BA.5 infection, the group that received three vaccine doses showed significantly higher NAb levels than the groups that received zero, one, or two doses (Fig. 2B). Furthermore, those who received two doses exhibited significantly higher levels of anti-BA.5 NAbs compared to those who were unvaccinated. However, no significant differences were observed between the two-dose and one-dose groups or between the one-dose and zero-dose groups. The effect of vaccine dose on NAb titer also varied significantly among the different variants. For the anti-WT NAb titer, a clear increase was observed at higher vaccination doses (3 > 2 > 1 > 0; Fig. 2B). The anti-XBB1.9.1 titer for the three-dose group was notably higher than that for the other doses, whereas no significant difference was observed between the two-, one-, and zero-dose groups (Fig. 2B). Notably, different anti-BA.5 NAb doses were only observed between the ≥2 doses and <2 doses at 3 months post-infection (Fig. 2C), indicating that ≥2 vaccine doses were necessary for long-lasting immunity.

**Fig 3:**
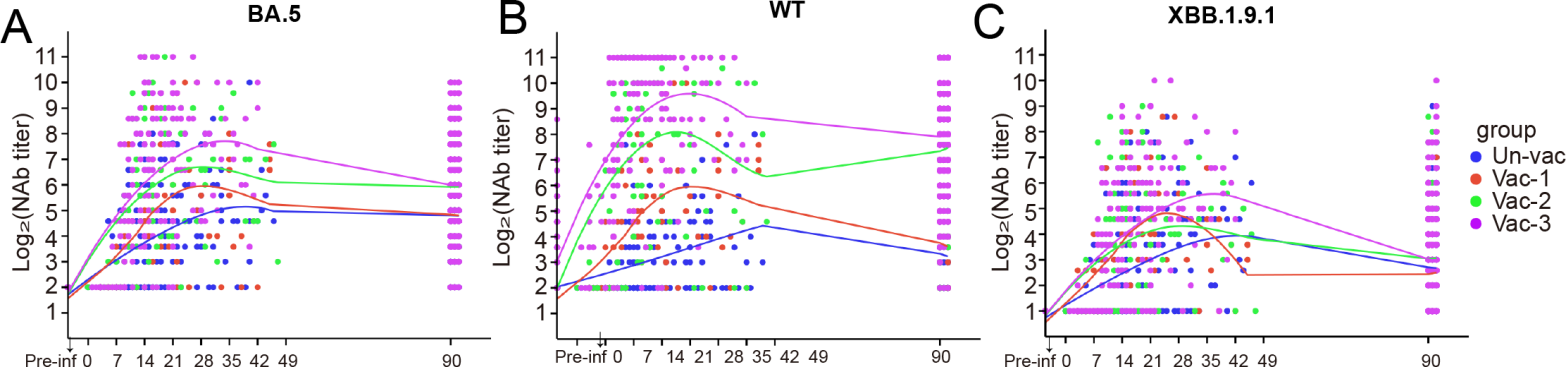
Neutralizing antibody titers fitted curves following initial infection in individuals with different immunization histories. (**A)** Fitted curves for NAbs against BA.5. (**B)** Fitted curves for NAbs against WT. (**C)** Fitted curves for NAbs against XBB.1.9.1.

To investigate whether vaccine doses affect the magnitude of NAbs against different strains and variants, as well as how rapidly they are generated, we used the ggplot2 package for R (version 4.2.1) to generate fitted curves based on NAb titers at certain time points. Our data revealed that anti-BA.5 NAbs in the two- or three-dose groups were induced earlier and at higher levels (within 1 week) vs those in the one- or zero-dose groups (Fig. 3A). A similar trend was observed for anti-WT NAbs (Fig. 3B) but not for anti-XBB1.9.1 NAbs (Fig. 3C).

To investigate the potential impact of the number of vaccination doses on immune imprinting following BA.5 infection, we calculated the ratios of anti-WT, anti-BA.5, and anti-XBB1.9.1 NAbs at 3 months following BA.5 infection, as an indicator of immune imprinting. The ratios in the patients who received three, two, one, and zero doses were 31.4:8.1:1, 20.2:7.3:1, 2.4:5.1:1, and 1.5:4.4:1, respectively (Fig. S1). Our findings clearly show that different numbers of vaccine doses have different impacts on immune imprinting following BA.5 infection, with higher doses of WT vaccines resulting in more dominant anti-WT immune imprinting.

We then conducted an analysis to assess the impacts of inactivated virus and S protein-based vaccines on NAb levels 3 months following infection. Our findings revealed no statistically significant difference in the anti-BA.5 or anti-WT immune responses elicited by these two vaccine types when administered in equivalent doses (Fig. S2A and B).

### Two or more WT vaccine doses reduced the risk of XBB reinfection further compared to one or no doses

**Fig 4:**
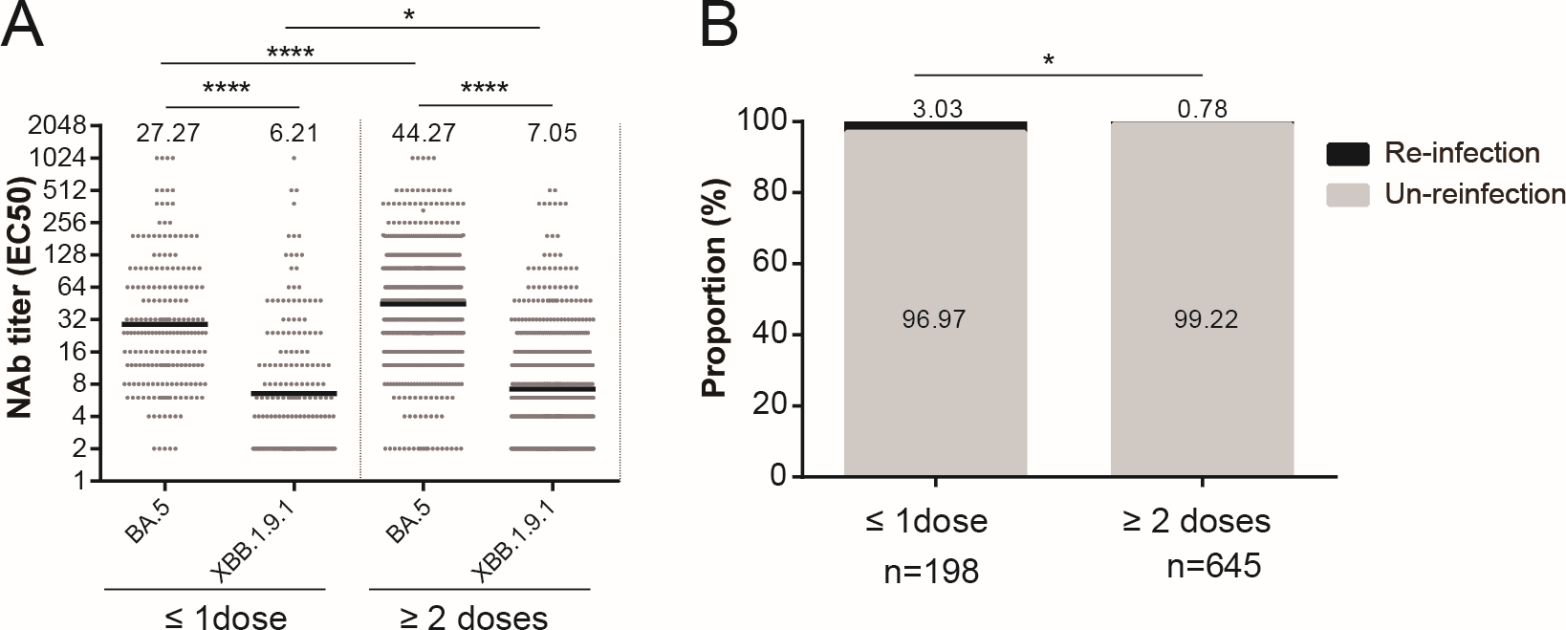
Administration of at least two vaccine doses effectively prevented the risk of reinfection. (**A)** Neutralizing antibody titers against BA.5 or XBB.1.9.1 among individuals who received ≥2 or ≤1 dose of a COVID-19 vaccine. (**B)** Reinfection rate among individuals who received ≥2 or ≤1 dose of a COVID-19 vaccine. Counts were analyzed using the Chi-squared test. The NAb titers in each group are presented as geometric mean titers at the top of each panel. Significance was determined using Mann-Whitney *U* tests. *P < 0.05, **P < 0.01, ***P < 0.001, and ****P < 0.0001.

To investigate the impact of WT vaccine doses on XBB variant reinfection risk, we conducted an observational study in a hospital during the XBB reinfection wave among 843 hospitalized patients over 1 week. Notably, none of the patients were infected with SARS-CoV-2 when they were admitted to the hospital. They had varying vaccination histories—including zero, one, two, three, and four vaccine doses—and had previously contracted BA.5 infections 5 months prior. Throughout the observation period, XBB reinfection was confirmed through polymerase chain reaction (PCR) testing. Upon hospital admission, we performed a serum test to measure NAbs against the BA.5 and XBB.1.9.1 variants. Our findings revealed that those who had received either zero or one dose had significantly lower NAb levels compared to those who had taken ≥2 doses (Fig. 4A). Notably, the rate of XBB reinfection over the 1-week observation period was significantly lower among those who had received ≥2 doses (5/645, 0.78%) compared to those who were either unvaccinated or had received only one dose (6/198, 3.03%, *P* = 0.014; Fig. 4B). These results underscored the effectiveness of administering ≥2 doses of the WT vaccine in terms of reducing the risk of XBB reinfection.

### Administration of two or three WT vaccine doses promoted viral clearance and attenuated the inflammatory response following BA.5 infection

**Fig 5:**
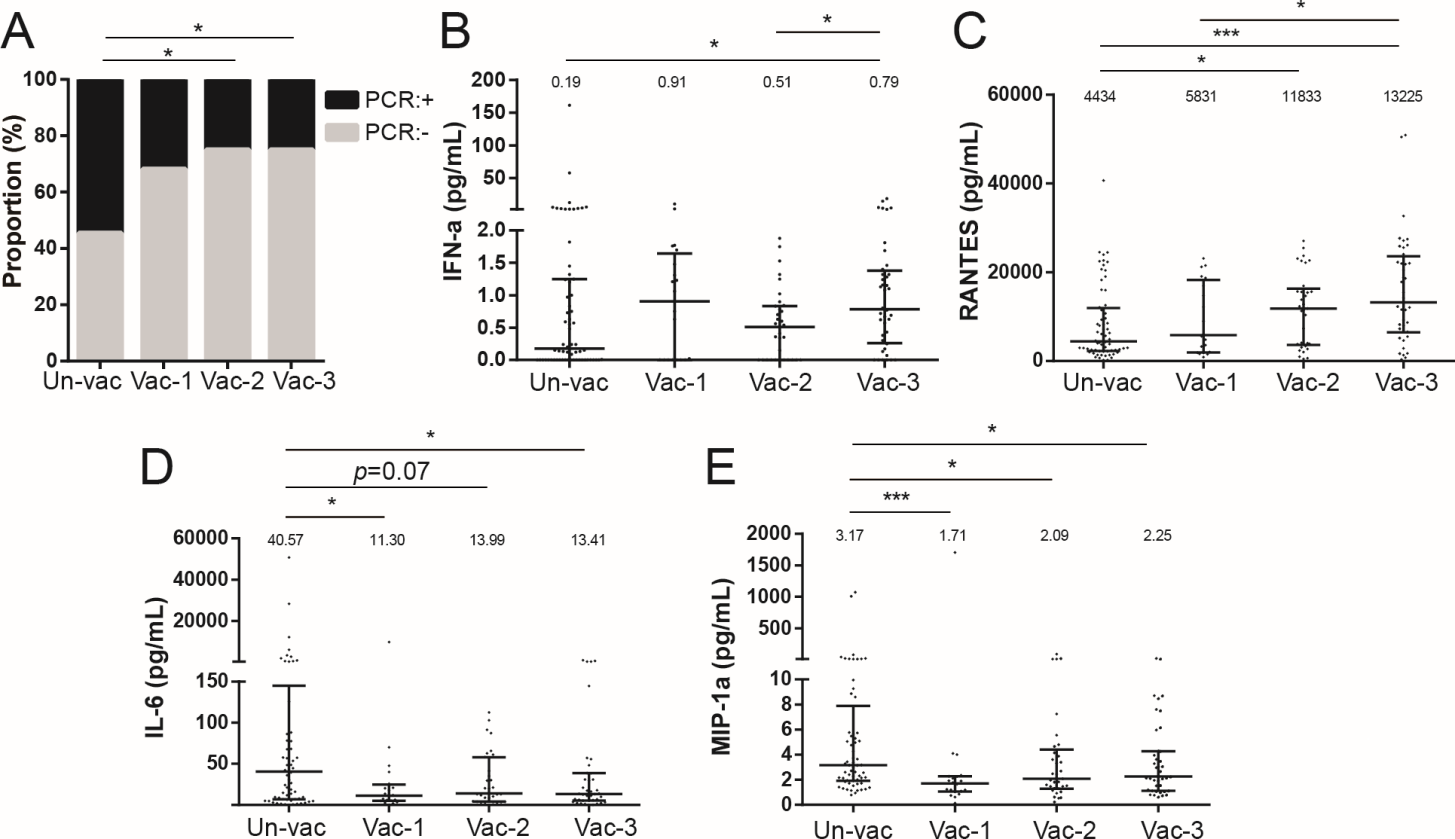
Proportion of negative nucleic acid conversion and cytokine profiles in patients vaccinated with zero, one, two, or three doses. (**A)** Proportion of negative nucleic acid conversion among different vaccination groups at 14 days post-infection. (**B)** IFN-α levels among the different groups. (**C)** RANTES levels among the different groups. (**D)** Interleukin (IL)-6 levels among the different groups. (**E)** Macrophage inflammatory protein (MIP)-1α levels among the different groups. Counts were analyzed using the Chi-squared test. The cytokine levels in each group are presented as median values at the top of each panel, and statistical significance was determined using Mann-Whitney *U* tests for the cytokines. *P < 0.05, **P < 0.01, ***P < 0.001, and ****P < 0.0001.

To investigate the impact of vaccine doses on viral clearance and the inflammatory response, SARS-CoV-2 nucleic acids and inflammation-associated cytokines were analyzed in different vaccination groups on the 14th day post-infection (Tables S1 and S2). The data revealed that those who had been vaccinated with two or three doses exhibited a significantly higher rate of negative nucleic acid conversion (>70%) than the unvaccinated population (45.45%) at 14 days after symptom onset (Fig. 5A). The levels of the antiviral cytokine interferon (IFN)-α were higher in the three-dose group compared to both the unvaccinated and two-dose groups (Fig. 5B). The concentration of Regulated upon Activation, Normal T Cell Expressed and Presumably Secreted (RANTES), a major human immunodeficiency virus-suppressive factor (19), exhibited a statistically significant increase in the two- and three-dose groups compared to the unvaccinated one (Fig. 5C). Furthermore, the levels of the proinflammatory factors IL-6 and MIP-1α were comparatively lower in the vaccinated populations compared to the unvaccinated ones (Fig. 5D and E). However, no significant differences were observed in terms of IL-2 and IL-1β levels among the three groups (Fig. S3A and B). These findings suggested that the administration of multiple WT vaccine doses effectively promoted viral clearance and attenuated the inflammatory response.

### Vaccination with two or three doses promoted B cell proliferation and differentiation, complement activation, and monocyte activation, while also reducing inflammation

**Fig 6:**
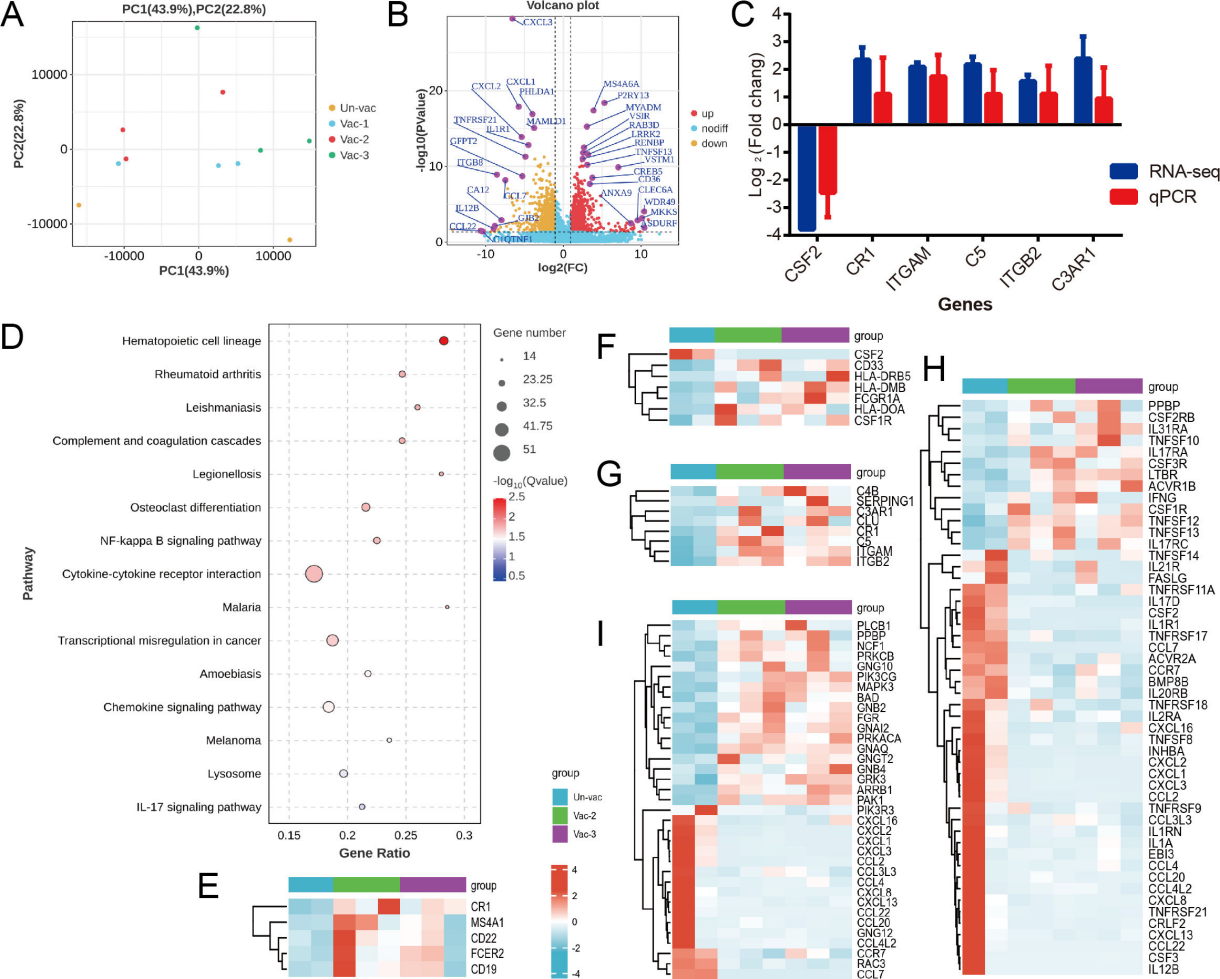
Gene expression signature of peripheral blood mononuclear cells between the two- or three-dose vaccinated group and the unvaccinated infection group. (**A)** Principal component analysis of the gene expression data computed for all of the genes from the different vaccination groups. (**B)** Volcano plot showing differentially expressed genes between the Vac-2/3 and Un-vac groups. (**C)** Validation of differentially expressed genes by quantitative real-time polymerase chain reaction. (**D)** Enriched Kyoto Encyclopedia of Genes and Genomes pathways among the differentially expressed genes between the Vac-2/3 and Un-vac groups. (**E)** Heatmap of significantly upregulated B cell surface molecule genes between the Vac-2/3 and Un-vac groups. (**F)** Heatmap of differentially expressed monocyte surface molecule genes between the Vac-2/3 and Un-vac groups. (**G)** Heatmap of differentially expressed complement pathway genes between the Vac-2/3 and Un-vac groups. (**H)** Heatmap of differentially expressed cytokine and chemokine genes between the Vac-2/3 and Un-vac groups. (**I)** Heatmap of differentially expressed chemokine genes between the Vac-2/3 and Un-vac groups.

To investigate the effect of vaccine doses on vaccination on the immunity induced by BA.5 infection at the molecular level, we performed RNA-seq analysis on peripheral blood mononuclear cell (PBMC) samples collected 14 days after symptom onset (Table S3). Principal component analysis revealed significant overall differences in the transcriptomes between the vaccinated (one, two, or three doses) and unvaccinated groups (Fig. 6A). The Vac-2 group could not be distinguished from the Vac-3 group. Compared to the Un-vac group, we observed the downregulation of 915 genes (fold change > 2, *P* < 0.05) and the upregulation of 1,030 genes in the Vac-2/3 group (Fig. 6B). Six genes were chosen for further validation by quantitative real-time PCR (qPCR). The results showed a concordant expression pattern with the RNA-seq data (Fig. 6C; Table S4), thereby confirming the reliability of the results. The differentially expressed genes (DEGs) we identified were predominantly enriched in various immune-related pathways, with the strongest enrichment being in the hematopoietic cell lineage (*P* = 1 × 10^−6^; Fig. 6D). The upregulation of genes encoding B cell surface molecules—including *CD19*, *MS4A1*, *CD22*, *FCER2*, and *CR1*—may be involved in the proliferation and differentiation of memory B cells induced by the vaccine doses, following BA.5 infection (Fig. 6E). The monocyte surface molecular genes *HLA-DOA*, *HLA-DMB*, *HLA-DRB5*, *CD33*, *FCGR1A*, and *CSF1R* were found to be upregulated in the Vac-2/3 group compared to the Un-vac one (Fig. 6F). By contrast, *CSF2* expression was downregulated (Fig. 6F). Changes in these genes may be involved in the regulation of monocyte activation induced by vaccination. Furthermore, the Vac-2/3 groups exhibited upregulation of the complement components C4, C5, C3AR1, CR3, and CR4, along with the upregulation of the negative regulatory factors C1INH, CR1, and Clusterin in the complement pathway. This suggested that vaccination with two or three doses may promote complement pathway activation under precise regulation (Fig. 6G). Meanwhile, a pattern of downregulation was identified for most genes enriched in cytokine-cytokine receptor interactions (Fig. 6H) and chemokine signaling pathways (Fig. 6I). Alterations in these genes may play a role in the attenuation of inflammation induced by two or three vaccine doses.

## DISCUSSION

**Fig 7:**
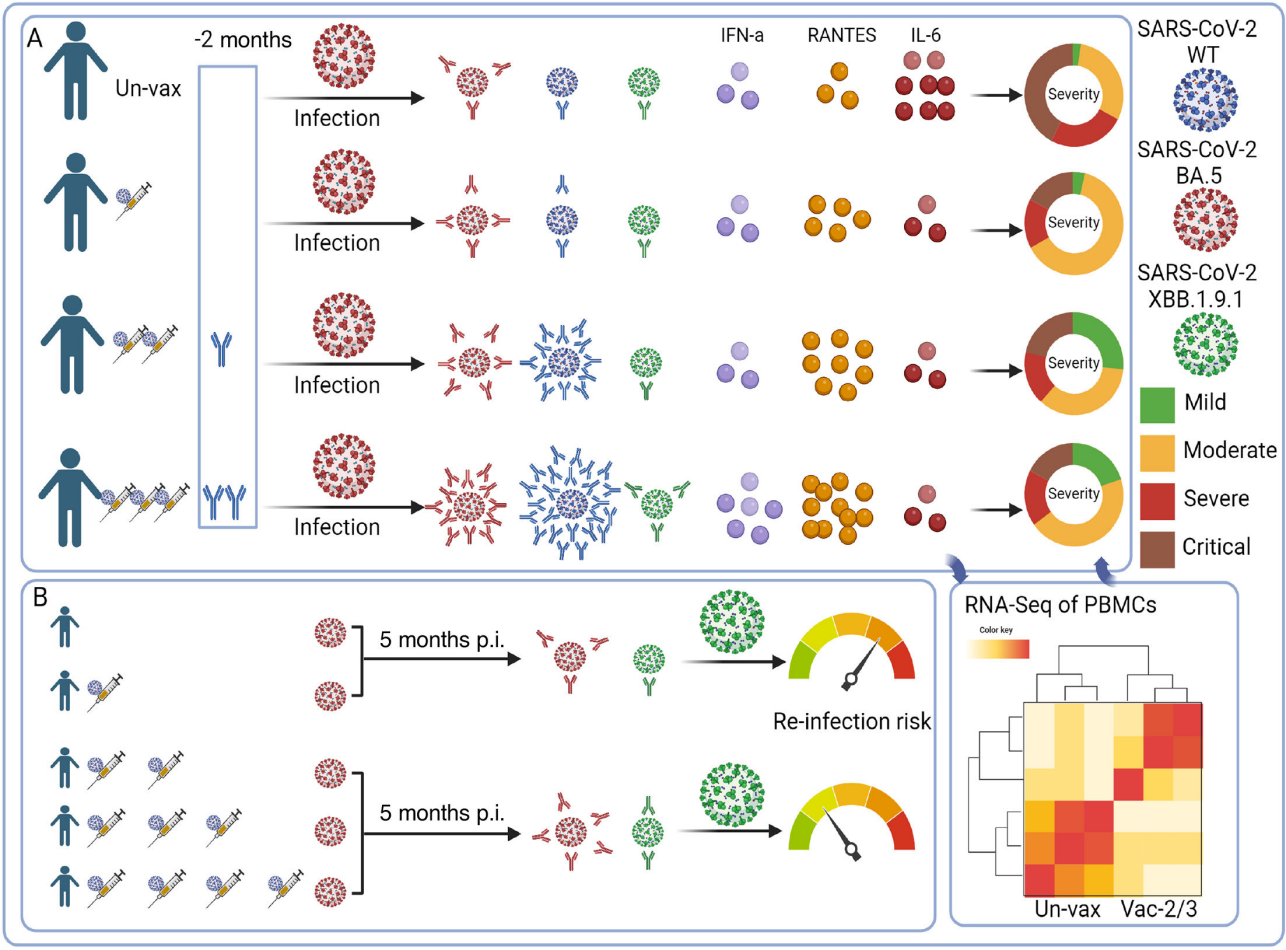
Schematic representation of the effect of wild-type vaccine doses on BA.5 hybrid immunity, disease severity, and XBB reinfection risk. (**A)** Representation of virus-specific immune effectors and the clinical severity during acute SARS-CoV-2 infection across vaccine doses. (**B)** The immune response and reinfection risk 5 months following initial COVID-19 development across vaccine dose groups. Created with Biorender.com.

The effects of varying COVID-19 vaccine doses have been extensively studied. Higher numbers of vaccine doses were reported to generate more NAbs against SARS-CoV-2 prior to the emergence of the BA.1 variant (20–23). Therefore, three doses can provide more protection against hospitalization and contribute to reduced fatality rates (24–28). By contrast, the WT vaccine was administered in varying doses (≤3) to billions of individuals but resulted in undetectable anti-BA.5 NAbs. With the emergence of the BA.5 variant in December 2022 in China, the effectiveness of vaccination status against BA.5 infection has been questioned, as well as its role in terms of affecting disease severity, post-infection immunity, viral clearance, and the increasing inflammation induced by higher viral loads. This study retrospectively demonstrated that, compared to a single dose or no vaccination, two or three doses of the WT SARS-CoV-2 vaccine induced higher levels of anti-BA.5 NAbs produced more quickly. This suggests more WT-dominant immune imprinting, increased facilitation of viral clearance, reduced viral inflammation, and attenuated disease severity under this vaccination regimen (Fig. 7A). At the molecular level, we confirmed that multiple vaccinations induced greater humoral immunity, including antibody and complement responses. Furthermore, the administration of ≥2 vaccine doses conferred a significant advantage in terms of mitigating the risk of reinfection with the XBB variant (Fig. 7B).

A rapid and heightened anti-BA.5 NAb response to SARS-CoV-2 infection should be considered a recall response that is induced by shared epitopes common to both the BA.5 and WT strains—leading to accelerated viral clearance and reduced viral inflammation. Our study provided an immunological explanation for real-world data showing that ≥2 doses of inactivated virus vaccine provided adequate protection against COVID-19-associated pneumonia (66%) and severe or critical COVID-19 (91%) (1). Furthermore, the finding that three doses generated more WT-dominant NAbs following BA.5 infection than zero, one, or two doses suggested that vaccine dosage influenced the immune imprinting shift even when administered >1 year after a previous dose that produced undetectable levels of anti-BA.5 NAbs. Our data partially support a previous hypothesis that the degree of imprinting may depend on specific variants, the order in which they are introduced in the individual’s immune system and the number of exposures (including the number of vaccine doses received) (29). Moreover, our neutralizing antibody data clearly demonstrated a significant impact on immune imprinting for mismatches between the strains involved in vaccination and infections, requiring ≥2 doses for effective protection across different strains (Fig. 2 and 3). This is further supported by our RNA-seq data, which indicated that ≥2 WT vaccine doses induced higher antibody levels, resulting in an enhanced antibody-dependent complement response and monocyte activation that alleviated inflammation. Theoretically, the optional number of doses required depends on the vaccine modality (such as inactivated, live attenuated, subunit, or mRNA vaccine), the nature of the antigen present, pre-existing immunity, the target population (such as older vs younger individuals), and the encountered variants (30). For example, a two-dose measles vaccine regimen in children provides lifelong protective immunity, whereas three to four doses of hepatitis B vaccine are necessary for adequate protection (31). Furthermore, the Advisory Committee on Immunization Practices recommends the annual administration of one to two doses of the updated influenza vaccine to enhance protective efficacy, owing to the continuous mutation of this virus. Our research revealed that the administration of ≥2 doses of either an inactivated SARS-CoV-2 virus vaccine or an S protein-based vaccine notably increased the generation of targeted neutralizing antibodies specific to the BA.5 variant, during infection with the strain. Notably, vaccination schedules involving two to three doses effectively expedited viral clearance, attenuated inflammatory responses, and clinically translated into reduced disease severity. For SARS-CoV-2 vaccination or infection, significant immune imprinting may require ≥2 encounters with the antigen, either against matched or mismatched variants, possibly owing to the inherent structural characteristics of the antigen.

A significant increase in immune evasion capabilities has been observed in the XBB recombinant of SARS-CoV-2 (32). Hybrid immunity elicited an elevated level of NAbs against the Omicron subvariants (33). Previous infection with BA.1, combined with primary series vaccination, has not been shown to provide significant protection against reinfection with XBB (34). By contrast, previous BA.1 infection accompanied by a booster vaccine dose or previous BA.2 infection along with primary series vaccination offered moderate protection (34). Notably, the combination of previous BA.2 infection and a booster vaccine dose demonstrated the highest level of protective efficacy (34). Although the present study was based on a small cohort of hospitalized patients (*n* = 843) over a very short observation period (1 week), we were able to demonstrate the impact of the vaccination doses on the risk of reinfection with the emerging XBB.1.9.1 strain. This may be influenced by anti-XBB.1.9.1 NAbs induced by shared epitopes between the WT and XBB, as well as BA.5 and XBB strains. In fact, at 20–50 days after BA.5 infection, the levels of anti-XBB NAbs induced by three vaccine doses (geometric mean titer, GMT 36.4) were significantly higher than those induced by other doses (GMT 12.1–15.7), while the difference in anti-XBB NAb levels waned among different doses at 3 months post-infection (GMT 5.5–8.3). This indicated that a more extensive B cell response was generated against XBB for three doses at 20–50 days following BA.5 infection, resulting in the differentiation of a stronger memory B response at 5 months post-infection—thereby resulting in a more rapid recall response upon XBB exposure (35). Notably, recent studies have revealed that specific serum antibody levels against the XBB variant do not represent a risk factor for contracting infections with the variant in patients with nasopharyngeal carcinoma and those who are on hemodialysis (36, 37). In fact, following 3 years of the COVID-19 pandemic with the threshold of protection against specific SARS-CoV-2 strains remaining not well defined, we found that NAb levels did not relate to recovery in cases of severe COVID-19 (38). This suggests that the host requires a certain level of NAbs to prevent viral infection or promote recovery from the disease—but that above this threshold level, more NAbs are not associated with protection against infection or reinfection. On the other hand, how quickly a patient can reach this NAb threshold upon reinfection may determine the disease severity. Our data showed that more vaccine doses can help vaccinated individuals produce NAbs quicker and at higher levels, which may help mitigate the occurrence and severity of reinfection. However, whether more vaccine doses can provide protection against subsequent variants following XBB infection remains unknown.

The rapid development and global deployment of COVID-19 vaccines in response to the pandemic are unprecedented, raising questions regarding vaccination status that pertain not only to dosing numbers and intervals but also its efficacy against different variants. Despite the limited number of circulating SARS-CoV-2 strains that have been observed, our study offers a unique perspective on the impact of varying vaccination regimens on the viral immunological response and subsequent reinfection risk by other variants, thereby providing guidance for future vaccination campaigns.

## MATERIALS AND METHODS

### Study design and participants

This study aimed to investigate the impact of WT SARS-CoV-2 vaccination schedules on the immune response and COVID-19 disease severity following BA.5 infection, as well as the risk of reinfection. All of the participants (*n* = 2,216) were recruited from the Guangzhou First People’s Hospital, and their basic information and vaccination records were obtained from the hospital’s medical records. Blood samples were systematically gathered from the participants during four distinct phases—the pre-infection period, the acute phase, and at 3 and 5 months post-infection—to facilitate comprehensive analyses across the disease course.

As of October 2022, 214 samples had been obtained from a cohort of vaccinated or unvaccinated individuals who had not previously contracted SARS-CoV-2. Among them, 29 had not received any COVID-19 vaccination and 17, 57, and 111 had received one, two, and three doses, respectively (Fig. 1A, pre-infection). During the BA.5 epidemic, a total of 592 samples were collected from 359 patients with COVID-19 confirmed using a SARS-CoV-2 PCR test administered at 0–50 days following symptom onset (Fig. 1A, acute phase). Among them, 106 had not received any COVID-19 vaccination and 68, 70, and 115 had received one, two, and three doses, respectively. Their samples were used to evaluate their titers of SARS-CoV-2-specific NAbs and cytokine profiles during the acute phase of the disease. Three months following infection, a total of 800 participants were analyzed—of whom 139 had received no COVID-19 vaccination and 34, 126, and 501 had received one, two, and three doses, respectively (Fig. 1A, 3 months p.i.). Convalescent specimens were used to track their waning NAb levels. During the subsequent XBB wave, 843 samples were collected from hospitalized patients without XBB variant infections—of whom 162 had received no COVID-19 vaccination and 36, 135, 492, and 18 had received one, two, three, and four doses, respectively (Fig. 1A, 5 months p.i.). We tracked their SARS-CoV-2 infection statuses during the XBB outbreak via PCR confirmation at the same hospital over a 1-week period.

COVID-19 severity was defined using the Diagnosis and Treatment Protocol for Novel Coronavirus Pneumonia (Trial Version 10) published by the National Health Commission of China. These definitions encompass (i) mild: symptomatic patients who meet the COVID-19 case definition but do not show signs of viral pneumonia or hypoxia; (ii) moderate: patients with pneumonia detected via imaging, respiratory rate of <30 breaths/min and pulse oxygen saturation (SpO_2_)of <93% on room air; (iii) severe: patient with pneumonia plus one of the following: progressive worsening of clinical symptoms and imaging tests showing a > 50% increase in pulmonary lesions within 24–48 h, arterial partial pressure of oxygen (PaO_2_)/fraction of inspired oxygen (FiO_2_) of ≤300 mmHg, respiratory rate of ≥30 breaths/min, or oxygen saturation ≤93% on room air; (iv) critical: patients with criteria for acute respiratory distress syndrome necessitating mechanical ventilation, septic shock, or multiple organ failure requiring intensive care unit monitoring and treatment.

### SARS-CoV-2 conventional neutralization assay

The SARS-CoV-2 conventional neutralization assay was performed according to a previously described protocol (39). Heat-inactivated plasma samples were initially tested at a dilution of 1:8 for the WT and BA.5 strains, whereas a dilution of 1:4 was used for XBB.1.9.1. Because of antibody degradation, the samples collected 5 months after infection were diluted 1:4 for the BA.5 and XBB.1.9.1 strains. Stepwise 1:2 dilution was then performed to obtain eight data points. The diluted samples were subsequently mixed with 50 µL of viral solution containing SARS-CoV-2 WT, BA.5, or XBB.1.9.1 variants. Following incubation at 37°C for 1 h, Vero E6 cells (1.2 × 10^4^; ATCC, USA) were added to the mixtures. The cells were then incubated at 37°C in a humidified environment with 5% CO_2_ for 4 days. The cytopathic effects were examined using a Celigo Imaging Cytometer (Nexcelom Bioscience, Lawrence, MA, USA). The presence or absence of cytopathic effect was determined through comparisons with positive and negative controls in each well. When the NAb titer fell below the limit of detection, we assigned an NAb titer of 50% inhibitory dilution (EC50) of four for specimens diluted at a ratio of 1:8, and one of two for those diluted 1:4.

### Curve fitting and heatmap visualization

The ggplot2 package for R was used to generate a fitting curve illustrating the NAb data across various vaccination schedules during the initial wave of infection.

### qPCR

Oropharyngeal swab samples were collected to measure viral RNA. The presence of viral RNA was detected using commercially available kits (Sansure Biotech, Changsha, China) targeting the *ORF1ab* and *N* genes, according to the manufacturer’s instructions. This detection method uses qPCR. A positive result was determined if the Ct value was <40, whereas a negative or undetermined outcome was assigned to Ct values of ≥40.

This method was also used to detect the mRNA expression levels of the *CSF2*, *CR1*, *ITGAM*, *C5*, *ITGB2*, and *C3AR1* PBMC genes. Total RNA was extracted using a Rapid RNA Extraction Kit (Goonie, Guangzhou, China). Subsequently, cDNA was obtained by reverse transcription using HiScript III RT SuperMix (Vazyme, Nanjing, China). The ChamQ Universal SYBR qPCR Master Mix (Vazyme, Nanjing, China) was then used to perform qPCR according to the manufacturer’s instructions, with the appropriate primers (Table S4). The reaction conditions were 95°C for 30 s, 95°C for 10 s, and 60°C for 30 s, over a total of 40 cycles. Relative quantitation was performed via the ΔΔCt method, and the expression of detectable genes was normalized to the 28S RNA reference gene.

### Cytometric bead array

Cytokine levels were assessed using a cytometric bead array (CBA; CBA Human Soluble Protein Flex Set System; BD Biosciences) on an LSR Fortessa instrument (BD Biosciences). Interleukin (IL)-6, IL-2, IL-1β, macrophage inflammatory protein-1α, and IFN-α levels were determined using a CBA assay. RANTES levels were measured via another CBA assay, using a 10-fold dilution. The IL-6 and RANTES levels were then re-evaluated using 100-fold dilutions when their concentrations exceeded the detection range. All of the experiments were performed according to the manufacturer’s instructions. Analyte signal intensities were determined relative to their corresponding standards, and absolute concentrations of individual analytes were calculated using BD FCAP Array Software (BD Biosciences).

### RNA sequencing

Approximately 1 million live PBMCs were thawed and dissolved in 1 mL of TRIzol (Invitrogen, USA). The manufacturer’s instructions were followed to extract total RNA from the PBMCs using the reagent. An RNA-seq transcriptome library was prepared using a Hieff NGS Ultima Dual-mode mRNA Library Prep Kit (Yeasen, China) using 1 µg of total RNA. The library was sequenced using a NovaSeq 6000 instrument (Illumina). Raw sequence reads were subjected to fastp version 0.18.0 for filtering, which entailed the removal of reads containing adapters or >10% unknown nucleotides, and the exclusion of low-quality reads comprising >50% bases with *Q*-values of ≤20. The short-read alignment tool Bowtie2 version 2.2.8 was used to map reads against the ribosomal RNA database, followed by the removal of rRNA-mapped reads. Quality-filtered reads were subsequently mapped to the human genome using HISAT 2.2.4, and the other parameters were set as the default. The mapped reads of each sample were assembled using StringTie version 1.3.1 via a reference-based approach. For each transcription region, a transcript per kilobase of the exon model per million mapped reads value was calculated to quantify its expression abundance and variations using RSEM software (40). Differential RNA expression analysis was performed using DESeq2 software between pairs of different groups (41). The genes and transcripts with *P* values of <0.05 and absolute fold changes of ≥2 were considered DEGs or differentially expressed transcripts.

### Statistical analysis

Statistical analyses were performed using SPSS version 20.0 and GraphPad Prism version 6.01. NAbs are reported as geometric mean titers. The Mann-Whitney *U* test was used to compare the central tendencies between pairs of groups. The Wilcoxon matched-pair signed-rank test was used to compare NAb levels between pairs of groups within the same cohort. The counts were analyzed using the Chi-squared test with a significance level set at *P* < 0.05 (*P < 0.05, **P < 0.01, ***P < 0.001, and ****P < 0.0001).

## Data Availability

The raw sequencing data from this study have been deposited in the Genome Sequence Archive in the Beijing Institute of Genomics BIG Data Center (https://ngdc.cncb.ac.cn/gsa), Chinese Academy of Sciences (GSA-Human: HRA007305).

## SUPPLEMENTAL MATERIAL

Supplemental material jvi.01285-24-s0001.docx Figures S1 to S3; Tables S1 to S4.

ASM does not own the copyrights to Supplemental Material that may be linked to, or accessed through, an article. The authors have granted ASM a non-exclusive, world-wide license to publish the Supplemental Material files. Please contact the corresponding author directly for reuse.
