# Supplementary material for: Effect of wild-type vaccine doses on BA.5 hybrid immunity, disease severity, and XBB reinfection risk
Source: J Virol. 2024 Nov 5;98(12):e01285-24. doi: 10.1128/jvi.01285-24 (PMC11650986; doi:10.1128/jvi.01285-24)
Supplement: Supplemental material — Figures S1 to S3; Tables S1 to S4. [file jvi.01285-24-s0001.docx]

**Supporting information**

**SUPPLEMENTAL FIGURES**

**Fig. S1**

**
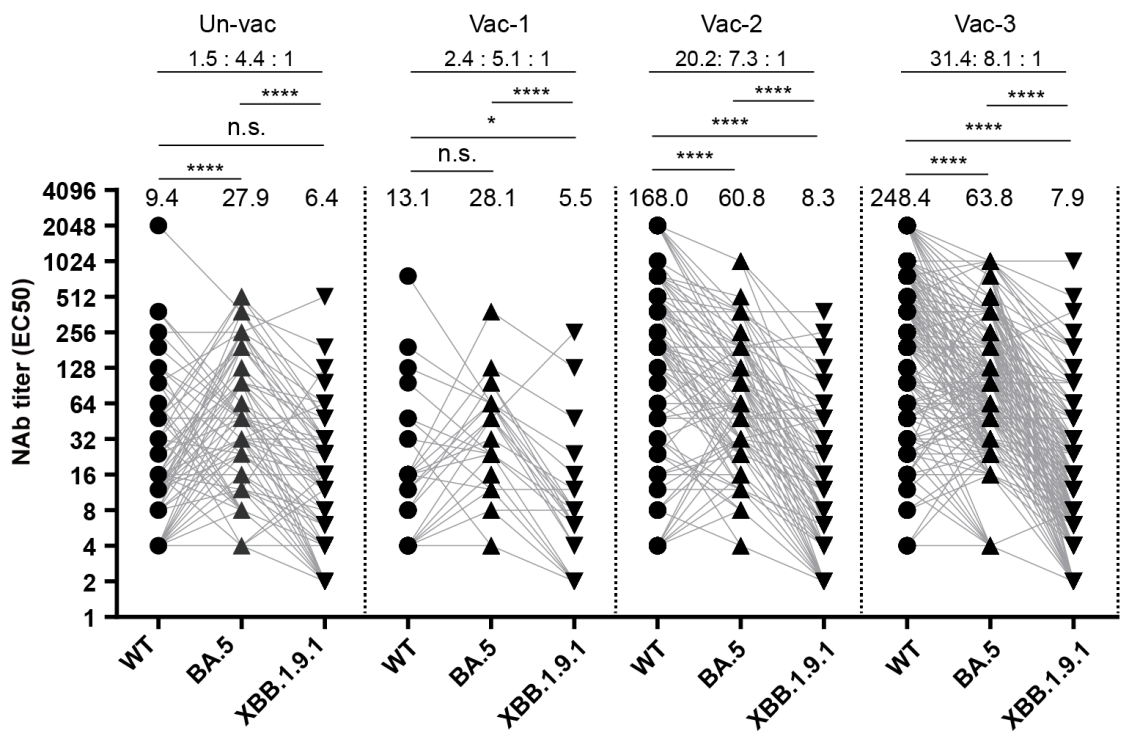
**

**Fig. S1. Ratio of anti-WT, anti-BA.5, and anti-XBB1.9.1 at 3 months following BA.5 infection as an indicator of immune imprinting.**

NAb titers against WT, BA.5, and XBB.1.9.1 were measured, and the ratios were compared to those against XBB.1.9.1. The NAb titers in each group are presented as geometric mean titers (GMTs) at the top of each panel. Significance was determined using Mann-Whitney U tests (**P*<0.05; ***P*<0.01; ****P*<0.001; *****P*<0.0001; and n.s., not significant.).

**Fig. S2**

**
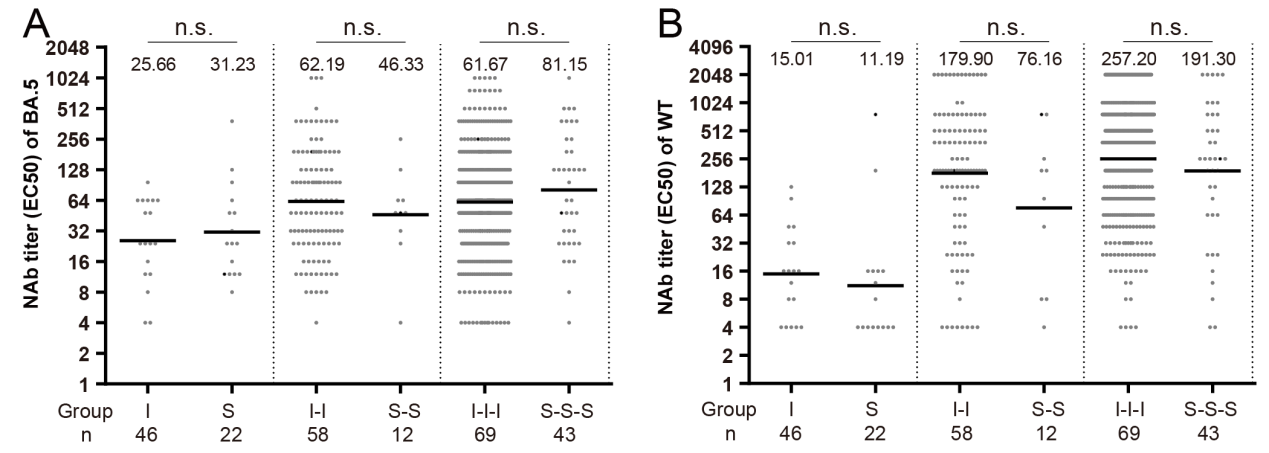
**

**Fig. S2. Neutralizing antibody (NAb) titers against BA.5 and WT in individuals vaccinated with 0, 1, 2, and 3 doses.**

A. NAb titers against BA.5 were assessed in individuals who received an inactivated virus vaccine, compared to those who received an S protein-based one.

B. NAb titers against WT were assessed in individuals who received an inactivated virus vaccine, compared to those who received an S protein-based one.

**Fig. S3**


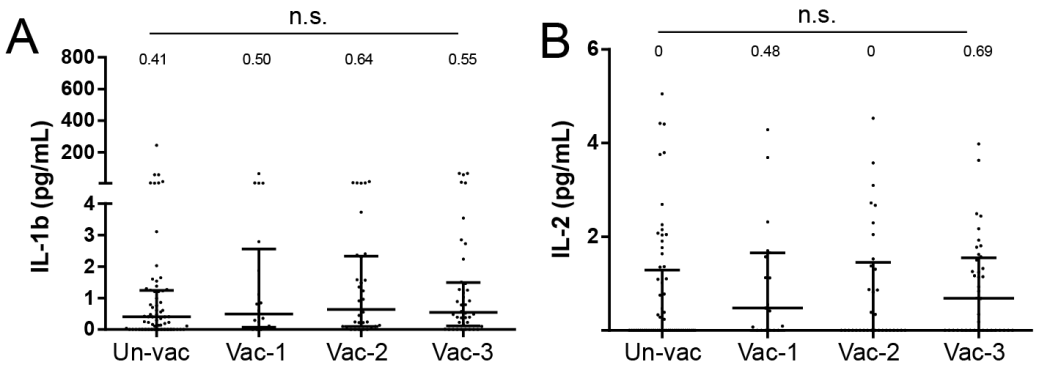


**Fig. S3. Interleukin (IL)-1β and IL-2 levels in individuals with different immune backgrounds.**

A. IL-1β levels across different groups.

B. IL-2 levels across different groups.

Counts were analyzed using the Chi-squared test. The cytokine levels in each group are presented as median values at the top of each panel, and significance was determined using Mann-Whitney U tests for the cytokines. **P*<0.05, ***P*<0.01, ****P*<0.001 and *****P*<0.0001.

**SUPPLEMENTAL TABLES**

**Table S1.** Demographics of COVID-19 patients who have the data of viral clearance.

| Group | Un-vac | Vac-1 | Vac-2 | Vac-3 |
| --- | --- | --- | --- | --- |
| n | 55 | 22 | 28 | 44 |
| Age-yr | | | | |
| Median (IQR) | 84.0 (72.0-89.0) | 80.5 (67.0-87.8) | 70.0 (48.5-83.8) | 75.0 (63.8-84.0) |
| Gender | | | | |
| Male | 38 (69.09) | 16 (72.73) | 16 (57.14) | 31 (70.45) |
| Female | 17 (30.91) | 6 (27.27) | 12 (42.86) | 13 (29.55) |
| Immunization strategies | | | | |
| I | / | 16 (72.73) | / | / |
| S | / | 6 (27.27) | / | / |
| I-I | / | / | 23 (82.14) | / |
| S-S | / | / | 5 (17.86) | / |
| I-I-I | / | / | / | 30 (68.18) |
| I-I-S | / | / | / | 1 (2.27) |
| S-S-S | / | / | / | 13 (29.55) |
| Severity | | | | |
| Mild | / | / | 4 (14.29) | 9 (20.45) |
| Moderate | 13 (23.64) | 10 (45.45) | 9 (32.14) | 14 (31.82) |
| Severe | 21 (38.18) | 5 (22.73) | 6 (21.42) | 13 (29.55) |
| Critical | 21 (38.18) | 7 (31.82) | 9 (32.14) | 8 (18.18) |

**Table S2.** Characteristics of COVID-19 patients who have been detected for cytokines.

| Group | Un-vac | Vac-1 | Vac-2 | Vac-3 |
| --- | --- | --- | --- | --- |
| n | 64 | 20 | 33 | 40 |
| Age-yr | | | | |
| Median (IQR) | 84.5 (74.5-90.0) | 70.5 (60.0-85.8) | 67.0 (48.0-74.50) | 69.50 (59.0-83.0) |
| Gender | | | | |
| Male | 42 (65.63) | 13 (65.00) | 19 (57.58) | 28 (70.00) |
| Female | 22 (34.37) | 7 (35.00) | 14 (42.42) | 12 (30.00) |
| Immunization strategies | | | | |
| I | / | 14 (70.00) | / | / |
| S | / | 6 (30.00) | / | / |
| I-I | / | / | 27 (81.82) | / |
| S-S | / | / | 6 (18.18) | / |
| I-I-I | / | / | / | 30 (75.00) |
| I-I-S | / | / | / | 2 (5.00) |
| S-S-S | / | / | / | 8 (20.00) |
| Severity | | | | |
| Mild | 1 (1.56) | 1 (5.00) | 7 (21.21) | 13 (32.50) |
| Moderate | 14 (21.88) | 10 (50.00) | 9 (27.27) | 10 (25.00) |
| Severe | 14 (21.88) | 3 (15.00) | 7 (21.21) | 10 (25.00) |
| Critical | 35 (54.69) | 6 (30.00) | 10 (30.30) | 7 (17.50) |

**Table S3** Characteristics of COVID-19 patients who have been detected by RNA-Seq.

| Group | Un-vac | Vac-1 | Vac-2 | Vac-3 |
| --- | --- | --- | --- | --- |
| n | 2 | 3 | 3 | 3 |
| Age-yr | | | | |
| Median (IQR) | 77.0 (70.0-840.0) | 53.0 (46.0-73.0) | 51.0 (35.0-67.0) | 63.0 (36.0-85.0) |
| Gender | | | | |
| Male | 1 (50.00) | 1 (33.33) | 2 (66.67) | 2 (66.67) |
| Female | 1 (50.00) | 2 (66.67) | 1 (33.33) | 1 (33.33) |
| Immunization strategies | | | | |
| I | / | 2 (66.66) | / | / |
| S | / | 1 (33.33) | / | / |
| I-I | / | / | 2 (66.66) | / |
| S-S | / | / | 1 (33.33) | / |
| I-I-I | / | / | / | 2 (66.66) |
| I-I-S | / | / | / | 1 (33.33) |
| Severity | | | | |
| Mild | / | 1 (33.33) | 2 (66.66) | 1 (33.33) |
| Moderate | 1 (50.00) | 2 (66.66) | / | 1 (33.33) |
| Severe | / | / | 1 (33.33) | 1 (33.33) |
| Critical | 1 (50.00) | / | / | / |

**Table S4** Primer sequences used in this study

| Primer ID | Sequence (5′−3′) |
| --- | --- |
| CSF2-F | GACCTCCAGGAGCCGAC |
| CSF2-R | TAATCTGGGTTGCACAGGAA |
| CR1-F | GGACAGGTGCAGACGTAAAT |
| CR1-R | GGCAGACGAGGAACCAATG |
| ITGAM-F | CCCTGGCCCATGTGAGAAC |
| ITGAM-R | AGCATATTTCACCGGCAGC |
| C5-F | CAGCGCGCTAGGATGC |
| C5-R | CTGAAATGACATATCTGCAACG |
| ITGB2-F | AGACTGGTAGCAAAGCCCCC |
| ITGB2-R | CACTCCTGAGAGAGGACGCAC |
| C3AR1-F | TAAGTGTGGGGACCAGACAG |
| C3AR1-R | AGACGCCATTGCTAAACAAA |
